# Genomic and pathogenic investigations of *Streptococcus suis* serotype 7 population derived from a human patient and pigs

**DOI:** 10.1080/22221751.2021.1988725

**Published:** 2021-10-17

**Authors:** Pujun Liang, Mingliu Wang, Marcelo Gottschalk, Ana I. Vela, April A. Estrada, Jianping Wang, Pengcheng Du, Ming Luo, Han Zheng, Zongfu Wu

**Affiliations:** a State Key Laboratory of Infectious Disease Prevention and Control, National Institute for Communicable Disease Control and Prevention, Chinese Center for Disease Control and Prevention, Beijing, People's Republic of China; b Guangxi Zhuang Autonomous Region Center for Disease Prevention and Control, Nanning, People's Republic of China; c Swine and Poultry Infectious Diseases Research Center, Faculty of Veterinary Medicine, University of Montreal, Canada; d Departamento de Sanidad Animal, Facultad de Veterinaria and Centro de Vigilancia Sanitaria Veterinaria (VISAVET), Universidad Complutense de Madrid, Madrid, Spain; e The College of Veterinary Medicine, University of Minnesota, St. Paul, MN, USA; f Beijing Key Laboratory of Emerging Infectious Diseases, Institute of Infectious Diseases, Beijing Ditan Hospital, Capital Medical University, Beijing, People's Republic of China; g Yulin Center for Disease Prevention and Control, Yulin, People's Republic of China; h OIE Reference Lab for Swine Streptococcosis, MOE Joint International Research Laboratory of Animal Health and Food Safety, College of Veterinary Medicine, Nanjing Agricultural University, Nanjing, People's Republic of China

**Keywords:** *Streptococcus suis* serotype 7, zoonotic pathogens, phylogeny, integrative mobilizable elements, virulence

## Abstract

*Streptococcus suis* is one of the important emerging zoonotic pathogens. Serotype 2 is most prevalent in patients worldwide. In the present study, we first isolated one *S. suis* serotype 7 strain GX69 from the blood culture of a patient with septicemia complicated with pneumonia in China. In order to deepen the understanding of *S. suis* serotype 7 population characteristics, we investigated the phylogenetic structure, genomic features, and virulence of *S. suis* serotype 7 population, including 35 strains and 79 genomes. Significant diversities were revealed in *S. suis* serotype 7 population, which were clustered into 22 sequence types (STs), five minimum core genome (MCG) groups, and six lineages. Lineages 1, 3a, and 6 were mainly constituted by genomes from Asia. Genomes of Lineages 2, 3b, and 5a were mainly from Northern America. Most of genomes from Europe (41/48) were clustered into Lineage 5b. In addition to strain GX69, 13 of 21 *S. suis* serotype 7 representative strains were classified as virulent strains using the C57BL/6 mouse model. Virulence-associated genes preferentially present in highly pathogenic *S. suis* serotype 2 strains were not suitable as virulence indicators for *S. suis* serotype 7 strains. Integrative mobilizable elements were widespread and may play a critical role in disseminating antibiotic resistance genes of *S. suis* serotype 7 strains. Our study confirmed *S. suis* serotype 7 is a non-negligible pathotype and deepened the understanding of the population structure of *S. suis* serotype 7, which provided valuable information for the improved surveillance of this serotype.

## Introduction

*Streptococcus suis* is an important emerging zoonotic pathogen responsible, among other infections, for septicemia, meningitis, endocarditis, and arthritis in humans [1]. To date, serotyping is an important routine diagnostic procedure and is widely used for subtyping *S. suis* strains. Among 29 confirmed serotypes (1–19, 21, 23–25, 27–31, and 1/2) and 28 novel *cps* types [2–5], serotype 2 is most frequently isolated from clinical cases in swine and humans worldwide [1,6]. Two outbreaks featured by high rates of streptococcal toxic-shock-like syndrome (STSLS) were caused by *S. suis* serotype 2 sequence type (ST)7 strains in China [7,8]. Recently, the prevalence of serotype 14 has also increased among sporadic human cases in China [9]. Serotype 9 has become the most prevalent serotype in diseased pigs in some European counties [1,10], and one human case of serotype 9 infection was reported [11]. Serotypes 4, 5, 16, 21, 24, and 31 have also been reported in human infections [1,12]. Serotype 7 is an important serotype frequently isolated from diseased pigs in European countries, North America, and Thailand [13–18], and it was also related to severe herd problems of meningitis and arthritis in nursery and grower pigs [14].

In the present study, an *S. suis* serotype 7 strain was isolated for the first time from the blood culture of a patient with septicemia complicated with pneumonia in China, suggesting that some serotype 7 strains may possess zoonotic potential. Except for limited epidemiologic studies [19] and *in vitro* survival assay in swine blood [20], little information is available for the phylogeny, evolution, and pathogenicity of *S. suis* serotype 7 population. The present study included 35 strains and 79 genomes of strains from 1999 to 2019 originating from nine countries to represent the *S. suis* serotype 7 population. The phylogenetic relationship, dissemination mechanisms of antibiotic resistance (AR) genes, variation of *cps* arrangements, and virulence were investigated to elucidate the population structure, genomic features, evolution, and pathogenicity of *S. suis* serotype 7.

## Materials and methods

### Case description

On 22 July 2016, a 71-year-old female patient with a history of hypertension was admitted to the First People’s Hospital of Yulin in Yulin city because of repeating fever and chill (highest body temperature of 39.5°C), cough, and abdominal pain for five days. A computerized tomography scan image indicated inflammation of both lungs. The serum level of high-sensitivity C-reactive protein and total counts of white blood cells were 112.77 mg/L and 5.68 × 10^9^/L, respectively. The neutrophil percentage was 72.7%. The patient’s blood pressure was 103/63 mm Hg. Meropenem, piperacillin/tazobactam, and levofloxacin were given as antibiotic therapy. The patient recovered and was discharged ten days later. A strain (named GX69) was isolated from the blood culture of the patient. The strain was confirmed as *S. suis* by amplifying *S. suis*-specific *recN* gene [21]. GX69 was first identified as serotype 7 by the agglutination test using the serum purchased from Statens Serum Institute, Copenhagen, Denmark, and further confirmed with a molecular serotyping method [22].

### Bacterial strains, genomes, and sequencing

For comparison purposes, 35 strains and 79 genomes were used in this study (Table 1). Twenty-seven of them were from China (24 of them were sequenced in the present study), 23 from the United States of American (USA), 22 from the United Kingdom (UK), 16 from Canada (sequenced in the present study), 13 from Spain (sequenced in the present study), 9 from the Netherlands (3 of them were sequenced in the present study), 2 from France (sequenced in the present study), 1 from Germany (sequenced in the present study) and Denmark each. Genomes of unspecified origin were from Genbank database. All genomes were re-confirmed to belong to *S. suis* by analysing their full length of 16s rRNA sequences [23] and *recN* gene specific to *S. suis* [24]. In addition, these genomes harboured *S. suis* serotype 7 specific *wzy* gene. They were isolated from 1999 to 2019.

**Table 1.** The information of strains and genomes used in the study.

Lineage	Name of Strain	MCG	Serotype	Sequence Type	*cps* subtype	Accession number	Host	Isolation source	Location	Year	AR genes
Lineage1	93.01B**^a^**	1	7	1609	7-II	SAMN17982935	Diseased pig	Heart	Spain	2001	*tet(W)*
	YS12	1	7	17	7-Ib	SAMN02469508	Healthy pig	Nasopharynx swab	CN	2012	*tet(O)*
	WUSS415**^a^**	1	7	1611	7–1	SAMN17982954	Healthy pig	Tonsil	CN	2017.12	*ant(6)-Ia, erm(B), tet(M)*
	WUSS417**^a^**	1	7	1611	7–1	SAMN17982955	Healthy pig	Tonsil	CN	2017.12	*ant(6)-Ia, erm(B), tet(M)*
Lineage2	2145959**^a^**	2	7	1613	7-1b	SAMN18117666	Diseased pig	Brain	Canada	2018	*tet(O), erm(B)*
	2255955**^a^**	2	7	89	7-5a	SAMN18117668	Diseased pig	Brain	Canada	2019	*tet(O), erm(B)*
	2120811**^a^**	2	7	89	7-Ia	SAMN18117667	Diseased pig	Liver	Canada	2018	*tet(O), erm(B)*
	2108284**^a^**	2	7	89	7-Ib	SAMN18117669	Diseased pig	Joint	Canada	2018	*tet(O), erm(B)*
	128.01B**^a^**	2	7	24	7-II	SAMN17982937	Diseased pig	Brain	Spain	2001	*tet(O),aph(3’)-IIIa,ant(6)-Ia,sat-4,erm(B)*
	255B**^a^**	2	7	24	7-II	SAMN17982942	Diseased pig	Lung	Spain	1999	*tet(O),aph(3’)-IIIa,ant(6)-Ia,sat-4,erm(B)*
	173B**^a^**	2	7	24	7-II	SAMN17982941	Diseased pig	Brain	Spain	1999	*tet(O),aph(3’)-IIIa,ant(6)-Ia,sat-4,erm(B)*
	126.01B**^a^**	2	7	24	7-II	SAMN17982936	Diseased pig	Brain	Spain	2001	*tet(O),aph(3’)-IIIa,ant(6)-Ia,sat-4,erm(B)*
Lineage3a	Ssuis120	3	7	373	7-Ib	SRR9123103	Diseased pig	Meninges	USA	2016	*tet(O)*
	Ssuis95	3	7	373	7-Ia	SRR9123148	Diseased pig	Joint	USA	2016	*tet(O)*
	2018WUSS020**^a^**	3	7	373	7-Ib	SAMN17982957	Healthy pig	Tonsil	CN	2018.09	*tet(O)*
	2018WUSS025**^a^**	3	7	373	7-Ia	SAMN17982958	Healthy pig	Tonsil	CN	2018.09	*tet(O)*
	2018WUSS017**^a^**	3	7	373	7-Ia	SAMN17982956	Healthy pig	Tonsil	CN	2018.09	*tet(O)*
	WUSS401**^a^**	3	7	373	7-Ia	SAMN17982952	Healthy pig	Tonsil	CN	2017.12	*tet(O), erm(B)*
	2019WUSS018**^a^**	3	7	373	7-Ib	SAMN17982962	Healthy pig	Tonsil	CN	2019.11	*tet(O), erm(B)*
	2019WUSS020**^a^**	3	7	373	7-Ib	SAMN17982964	Healthy pig	Nasopharynx swab	CN	2019.11	*tet(O), erm(B)*
	2019WUSS019**^a^**	3	7	373	7-Ib	SAMN17982963	Healthy pig	Tonsil	CN	2019.11	*tet(O), erm(B)*
	2019WUSS017**^a^**	3	7	373	7-Ib	SAMN17982961	Healthy pig	Tonsil	CN	2019.11	*tet(O), erm(B)*
	GX69**^a^**	3	7	373	7-Ia	SAMN18029937	Patient	Blood	CN	2016	*tet(O), erm(B)*
	WUSS255**^a^**	3	7	373	7-Ib	SAMN17982946	Healthy pig	Nasopharynx swab	CN	2017.10	*tet(O), erm(B)*
	WUSS382**^a^**	3	7	373	7-Ia	SAMN17982951	Healthy pig	Tonsil	CN	2017.12	*tet(O), erm(B)*
	WUSS318**^a^**	3	7	373	7-Ia	SAMN17982949	Healthy pig	Tonsil	CN	2017.12	*tet(O), erm(B)*
	WUSS316**^a^**	3	7	373	7-Ia	SAMN17982948	Healthy pig	Tonsil	CN	2017.12	*tet(O), erm(B)*
Lineage3b	Ssuis359	3	7	373	7-Ia	SAMN11854340	Diseased pig	Lung	USA	2017	*tet(O), erm(B)*
	Ssuis93	3	7	94	7-Ia	SRR9123171	Diseased pig	Brain	USA	2016	*tet(O)*
	2156696**^a^**	3	7	94	7-Ia	SAMN18117674	Diseased pig	brain	Canada	2018	*–*
	Ssuis51	3	7	980	7-III	SRR9123229	Diseased pig	Lung	USA	2015	*tet(O), erm(B)*
	Ssuis118	3	7	979	7-Ia	SRR9123101	Diseased pig	Brain	USA	2016	*tet(O), erm(B)*
	Ssuis98	3	7	94	7-Ia	SRR9123145	Diseased pig	Brain	USA	2015	*erm(B), tet(O)*
	Ssuis39	3	7	94	7-III	SRR9123191	Diseased pig	Brain	USA	2015	*tet(O), erm(B)*
	Ssuis40	3	7	94	7-Ia	SRR9123184	Diseased pig	Meninges	USA	2015	*tet(O), erm(B)*
	2130772**^a^**	3	7	839	7-Ia	SAMN18117675	Diseased pig	Brain	Canada	2018	*tet(O), erm(B)*
	Ssuis45	3	7	94	7-Ia	SRR9123062	Diseased pig	Brain	USA	2015	*–*
	Ssuis109	3	7	94	7-Ia	SRR9123073	Diseased pig	Brain	USA	2015	*erm(B)*
	Ssuis303	3	7	94	7-III	SRR9123157	Diseased pig	Joint	USA	2016	*–*
	Ssuis41	3	7	94	7-Ia	SRR9123183	Diseased pig	Brain	USA	2015	*erm(B)*
	Ssuis46	3	7	94	7-III	SRR9123063	Diseased pig	Lung	USA	2014	*tet(O)*
	Ssuis324	3	7	94	7-III	SRR9123265	Diseased pig	Brain	USA	2017	*–*
	Ssuis108	3	7	94	7-Ia	SRR9123068	Diseased pig	Brain	USA	2015	*erm(B)*
Lineage4	WUSS004**^a^**	4	7	225	7-Ib	SAMN17982943	Diseased pig	/	CN	2016	*tet(O), erm(B)*
	WUSS029**^a^**	4	7	225	7-Ib	SAMN17982945	Healthy pig	/	CN	unknown	*tet(O), erm(B)*
	Ssuis8	4	7	225	7-Ib	SRR9123237	Diseased pig	Brain	USA	2014	*aph(3’)-IIIa,ant(6)-Ia, sat-4, erm(B), tet(O)*
	Ssuis11	4	7	225	7-Ib	SRR9123252	Diseased pig	Brain	USA	2014	*ant(6)-Ia(2),ant(9)-Ia, aph(3’)-IIIa, sat-4, erm(B), tet(O)*
	2234142**^a^**	4	7	1614	7-Ib	SAMN18117670	Diseased pig	Brain	Canada	2019	*erm(B)*
	WUSS013**^a^**	4	7	225	7-Ib	SAMN17982944	Diseased pig	/	CN	unknown	*tet(O), erm(B)*
	Ssuis100	4	7	225	7-Ib	SRR9123151	Diseased pig	Meninges	USA	2015	*tet(O)*
	2225102**^a^**	4	7	32	7-Ib	SAMN18117671	Diseased pig	Heart	Canada	2019	*tet(O), erm(B)*
	YS63**^a^**	4	7	32	7-Ia	SAMN20087851	Healthy pig	Nasopharynx swab	CN	2012	*tet(O),erm(B)*
	YS66	4	7	32	7-Ia	SAMN02469560	Healthy pig	Nasopharynx swab	CN	2012	*tet(O),erm(B)*
	2211488**^a^**	4	7	34	7-Ia	SAMN18117672	Diseased pig	Heart	Canada	2019	*tet(O), erm(B)*
	S12R	4	7	907	7-Ia	SAMEA3233991	Diseased pig	Lung	UK	2010	*–*
	SS1022	4	7	907	7-Ia	SAMEA1316674	Pig	/	UK	2014	*–*
	2135990**^a^**	4	7	32	7-Ib	SAMN18117673	Diseased pig	Brain	Canada	2018	*tet(O), erm(B)*
Lineage5a	2139811**^a^**	4	7	1610	7-Ia	SAMN18117676	Diseased pig	Brain	Canada	2018	*tet(O), erm(B)*
	2288194**^a^**	4	7	1610	7-Ia	SAMN18117677	Diseased pig	Brain	Canada	2019	*tet(O), erm(B)*
	2175452**^a^**	4	7	971	7-Ia	SAMN18117678	Diseased pig	Abdominal liquid	Canada	2019	*tet(O), erm(B)*
	2274226**^a^**	4	7	971	7-Ia	SAMN18117679	Diseased pig	Brain	Canada	2019	*tet(O), erm(B)*
	Ssuis265	4	7	971	7-Ia	SRR9123206	Healthy pig	Tonsil	USA	2016	*tet(O), erm(B)*
Lineage5b	2207481**^a^**	4	7	971	7-Ia	SAMN18117680	Diseased pig	Lung	Canada	2019	*tet(O), erm(B)*
	GD-0031	4	7	29	7-Ia	SAMEA3595225	Diseased pig	CSF	Netherlands	2002	*erm(B),tet(O)*
	GD-0067	4	7	29	7-Ia	SAMEA3595239	Diseased pig	CSF	Netherlands	2004	*erm(B),tet(O)*
	GD-0061	4	7	854	7-Ia	SAMEA3595236	Diseased pig	CSF	Netherlands	2004	*dfrF,ant(6)-Ia, tet(O), cat-TC*
	2138579**^a^**	4	7	29	7-Ib	SAMN18117681	Diseased pig	Brain	Germany	2018	*tet(O), erm(B)*
	2180644**^a^**	4	7	29	7-Ia	SAMN18117682	Diseased pig	Brain	Spain	2019	*tet(O)*
	WUSS366**^a^**	4	7	29	7-Ib	SAMN17982950	Healthy pig	Tonsil	CN	2017.12	*–*
	WUSS302**^a^**	4	7	29	7-Ia	SAMN17982947	Healthy pig	Tonsil	CN	2017.11	*tet(O), erm(B)*
	S11O	4	7	29	7-Ia	SAMEA3233988	Diseased pig	Lung	UK	2010	*lnu(B), aph(3’)-IIIa, lsaC,ant(9)-Ia*
	SS1018	4	7	29	7-Ia	SAMEA1316697	Pig	/	UK	2014	*aph(3’)-IIIa, lnu(B), lsaC,ant(9)-Ia*
	Ssuis77	4	7	973	7-Ib	SRR9123095	Pathogenic	Liver	USA	2014	*erm(B)*
	2243014**^a^**	4	7	29	7-Ia	SAMN18117684	Diseased pig	Brain	Canada	2019	*tet(O), erm(B)*
	2114366**^a^**	4	7	29	7-Ib	SAMN18117683	Diseased pig	Joint	France	2018	*aph(3’)-IIIa,lnu(B),lsaC,ant(9)-Ia*
	S10W	4	7	29	7-Ia	SAMEA3233977	Diseased pig	Lung	UK	2010	*tet(W),erm(B)*
	Ssuis139	4	7	29	7-Ia	SRR9123173	Diseased pig	Joint	USA	2016	*tet(O), erm(B)*
	Ssuis136	4	7	29	7-Ib	SRR9123182	Diseased pig	Meninges	USA	2016	*tet(O), erm(B)*
	SS1000	4	7	29	7-Ia	SAMEA1316594	Pig	/	UK	2014	*tet(W),erm(B)*
	SS1051	4	7	29	7-Ib	SAMEA1316581	Pig	/	UK	2014	*erm(B),tet(O)*
	S15R	4	7	29	7-Ia	SAMEA3234014	Diseased pig	Brain	UK	2010	*erm(B),tet(O)*
	2148719**^a^**	4	7	29	7-Ia	SAMN18117685	Diseased pig	Spleen	Netherlands	2018	*tet(O), erm(B)*
	149B**^a^**	4	7	29	7-Ia	SAMN17982938	Diseased pig	Heart	Spain	1999	*–*
	LS4E	4	7	29	7-II	SAMEA3233917	Healthy pig	Tonsil	UK	2011	*dfrF,tet(M)*
	GD0094	4	7	29	7-Ib	SAMEA3595252	Diseased pig	CSF	Netherlands	2006	*–*
	12V457	4	7	29	7-Ib	SAMEA3595206	Diseased pig	CSF	Netherlands	2006	*erm(B),tet(O)*
	LS6Z	4	7	29	7-II	SAMEA3233938	pig	/	UK	2011	*dfrF,tet(M)*
	150B**^a^**	4	7	29	7-Ia	SAMN17982939	Diseased pig	Lymphatic gland	Spain	1999	*–*
	D9	4	7	29	7-Ib	SAMN02603321	Pig	/	CN	/	*erm(B),tet(O)*
	LSS83	4	7	29	7-II	SAMEA1316699	Pig	/	UK	2014	*dfrF,tet(M)*
	2114361**^a^**	4	7	29	7-Ia	SAMN18117687	Diseased pig	Brain	Spain	2018	*tet(O), erm(B)*
	2245605**^a^**	4	7	29	7-Ia	SAMN18117686	Diseased pig	Joint	Spain	2019	*tet(O), erm(B)*
	2245604**^a^**	4	7	29	7-Ia	SAMN18117688	Diseased pig	Spleen	Netherlands	2019	*tet(O), erm(B)*
	S10D	4	7	29	7-II	SAMEA3233974	Diseased pig	Brain	UK	2010	*tet(M)*
	LOLA-SS008	4	7	29	7-II	SAMEA1316648	Pig	/	UK	2014	*dfrF,tet(M)*
	2260249**^a^**	4	7	29	7-II	SAMN18117689	Diseased pig	Joint	Netherlands	2019	*tet(O), erm(B)*
	LS5F	4	7	29	7-II	SAMEA3233926	Healthy pig	Tonsil	UK	2011	*dfrF,tet(M)*
	GD-0070	4	7	29	7-Ib	SAMEA3595240	Diseased pig	CSF	Netherlands	2005	*erm(B),tet(O)*
	LSS85	4	7	29	7-II	SAMEA1316680	Pig	/	UK	2014	*dfrF,tet(M)*
	LL-W	4	7	29	7-II	SAMEA3233877	Diseased pig	/	UK	2010	*dfrF,tet(M)*
	LS3D	4	7	29	7-II	SAMEA3233910	Healthy pig	Tonsil	UK	2011	*dfrF,tet(M)*
	SS1007	4	7	29	7-II	SAMEA1316618	Pig	/	UK	2014	*tet(M)*
	LSS84	4	7	29	7-II	SAMEA1316689	Pig	/	UK	2014	*dfrF,tet(M)*
	2138581**^a^**	4	7	29	7-Ia	SAMN18117690	Diseased pig	Spleen	France	2018	*tet(O), erm(B)*
	8074	4	7	29	7-Ia	SAMN02469536	Diseased pig	/	Danmark	1980'	*–*
	2138556**^a^**	4	7	29	7-Ia	SAMN18117691	Diseased pig	Brain	Spain	2018	*–*
	LSS56	4	7	29	7-II	SAMEA1316549	Pig	/	UK	2014	*dfrF,tet(M)*
	2270437**^a^**	4	7	29	7-II	SAMN18117692	Diseased pig	Brain	Spain	2019	*tet(O), erm(B)*
	151B**^a^**	4	7	29	7-Ia	SAMN17982940	Diseased pig	Heart	Spain	1999	*–*
	LOLA-SS009	4	7	29	7-II	SAMEA1316611	Pig	/	UK	2014	*tet(M)*
	LL-X	4	7	29	7-II	SAMEA3233878	Diseased pig	Brain	UK	2010	*tet(M)*
	WUSS413**^a^**	4	7	29	7-Ib	SAMN17982953	Healthy pig	Tonsil	CN	2017.12	*tet(O), erm(B)*
Lineage6	2018WUSS100**^a^**	7–2	7	1612	7-III	SAMN17982959	Healthy pig	Tonsil	CN	2019.01	*ant(6)-Ia, aac(6′)-Ie-aph(2″)-Ia, erm(B), tet(M)*
	2018WUSS101**^a^**	7–2	7	1612	7-III	SUB9103358	Healthy pig	Tonsil	CN	2019.01	*ant(6)-Ia, aac(6′)-Ie-aph(2″)-Ia, erm(B), tet(M)*

^a^ genomes were sequenced in the present study. Non-indicated genomes from NCBI.

/: not available; –: None of AR genes

In the present study, the complete genome of strain GX69 was sequenced using PacBio Sequel platform and Illumina NovaSeq PE150, whereas the draft genomes were sequenced using Illumina NovaSeq PE150. Sequencing libraries were generated using the methods described previously [25]. The valid reads filtered low-quality reads were assembled into contigs and scaffolds with SOAP*denovo* (release 1.04). Genes were predicted by using Glimmer 3.02, and gene orthologs were determined by using GO (Gene ontology) V20171011, KEGG (Kyoto Encyclopedia of Gene and Genmomes) V20181107, and COG (Clusters of Othologous Database) V20171127.

### Bioinformatics analysis

#### MLST and MCG typing

The multilocus sequence type (MLST) and the minimum core genome (MCG) group of the genomes were determined by using PubMLST (https://pubmlst.org/bigsdb?db=pubmlst_ssuis_seqdef&page=sequenceQuery), and a method previously described [26], respectively.

#### Phylogenetic analysis

Single-nucleotide polymorphisms (SNPs) were detected using Bowtie 2, and MUMmer v3.23 for sequencing reads and complete genomes, respectively, and the genome sequence of SC84 (accession No. FM252031) [27] was used as a reference. The mutational SNP sites were selected based on the method described in a previous study [26], and then the phylogenetic tree was constructed using the maximum likelihood method by FastTree v2.1.10. *Streptococcus pneumoniae* ATCC 700669 (accession No. NC_011900) was used as an outgroup to root the tree. The tree was presented using FigTree v1.4.0.

#### Detection of S. suis virulence-associated genes, AR genes and AR genes associated with mobile genetic elements (MGEs)

Distributions of virulence-associated genes and regions of difference (RDs) preferentially present in highly pathogenic *S. suis* serotype 2 strains were investigated among *S. suis* serotype 7 genomes, consisting of genes *mrp*, *sly*, *epf*, *sao*, *nadR*, *NisR*, *NisK*, *SalR*, *SalK, revS*, *ofs*, RD6, RD12, RD14, RD21, RD29, RD40, RD53, and RD60 [28,29]. Genes having a global match region at <80% of the amino-acid sequence with an identity of <80% were determined to be absent.

AR genes were analysed by searching Comprehensive Antibiotic Resistance database (CARD) and Antibiotic Resistance genes database (ARDB). A resistance gene was only regarded as a homologue in tested strains if it showed at least 80% identity in amino-acid sequence across 80% of the length of the protein [30]. The prophages and ICEs were predicted by PHAST (http://phast.wishartlab.com/) and ICEberg (https://db-mml.sjtu.edu.cn/ICEberg/), respectively. For the identification of integrative and conjugative elements (ICEs), signature proteins including integrase, relaxase, and VirB4 were typed using the database from a previous study [31]. Search strategies and the definitions of integrative mobilizable elements (IMEs) and cis-IMEs (CIMEs) were carried out according to the methods previously described [31,32].

#### Analysis of *cps* gene cluster

Each *cps* gene cluster was extracted from the genomes and compared with that of the serotype 7 reference strain 8074 (GenBank accession No. BR001004.1). The homology groups (HGs) of *cps* genes were assigned according to the nomenclature described in a previous study [33]. The sequence comparison of *cps* gene cluster was performed using blastN programme in BLAST with an e-value cutoff of e-10 and was visualized using an in-house Perl script (https://github.com/dupengcheng/BlastViewer).

### Infection experiments

The virulence of strain GX69 from the patient and 21 additional representative strains based on the distribution in the phylogenetic tree were tested. For comparison, the highly pathogenic and well-characterized *S. suis* serotype 2 reference strain P1/7 (ST1) [34] was included [35,36]. C57BL/6 mice (6 weeks old, female) were injected intraperitoneally with 5 × 10^7^ CFU of *S. suis* strain in 1 mL PBS or 1 mL PBS only as a control group. The infection dose of each strain was confirmed by plating the serial dilutions of the suspension onto the Todd–Hewitt broth (THB, Oxoid Ltd, London, UK) agar before and after the infection. Each infected group contained ten mice, and the mock-infected group contained five mice. The mortality was recorded per six hours within 24 h post-infection and per 12 h from 24 h to 96 h post-infection. The experiment was performed independently at least twice for each strain. The mortality of each infected group was calculated via the Kaplan–Meier method. *S. suis* serotype 7 strains initiating lethal infection with a mortality ≥80% at 96 h post-infection were classified as virulent strains.

### Investigation of antimicrobial susceptibility profiles

To determine whether the AR genes in genomes conferred the predicted resistance to the corresponding bacteria, we used the MIC-test strip (Liofilchem, Roseto degli Abruzzi, Italy) to assess the antimicrobial susceptibility of strains carrying AR genes. The following antibiotics were tested: clindamycin (0.016–56 μg/mL), erythromycin (0.016–256 μg/mL), azithromycin (0.016–256 μg/mL), tetracycline (0.016–256 μg/mL), gentamicin (0.016–256 μg/mL), kanamycin (0.016–256 μg/mL), and streptomycin (0.064–1024 μg/mL). For tetracycline, azithromycin, erythromycin, and clindamycin, breakpoints were used as recommended by the Clinical and Laboratory Standard Institute (CLSI) guidelines 2019 (M100-S29) for *Streptococcus* spp. *Viridans* group. No breakpoint values of streptomycin, kanamycin, and gentamicin were available for *Streptococcus*. Their breakpoints were taken from a previous study [37].

### Statistics

The survival curves of different infected groups were compared using Gehan–Breslow–Wilcoxon test. For the test, a *p*-value < .05 was considered to be significant.

### Nucleotide sequence accession numbers

The sequences of the genomes sequenced in the study were deposited in the GenBank under accession numbers listed in Table 1.

### Ethical approval

This study and the application of the animal experiments with code 2020-024 were reviewed and approved by the ethics committee of the National Institute for Communicable Disease Control and Prevention, Chinese Center for Disease Control and Prevention.

## Results

### MLST and MCG typing

Among 114 genomes, 22 different STs were identified, revealing high heterogeneity of *S. suis* serotype 7 population. ST29 (*n* = 47) was most prevalent, followed by ST373 (*n* = 16), ST94 (*n* = 12), ST225 (*n* = 6), ST24 (*n* = 4), ST32 (*n* = 4), ST971 (*n* = 4), ST89 (*n* = 3), ST907 (*n* = 2), ST1610 (*n* = 2), ST1611 (*n* = 2), and ST1612 (*n* = 2). The remaining ST17, ST34, ST839, ST854, ST973, ST979, ST980, ST1609, ST1613, and ST1614 only contained one strain each. The strain GX69 from the patient was ST373 which was prevalent in China, whereas ST29 and ST94 were predominant in Europe and North America, respectively (Table 1).

**Figure 1.**
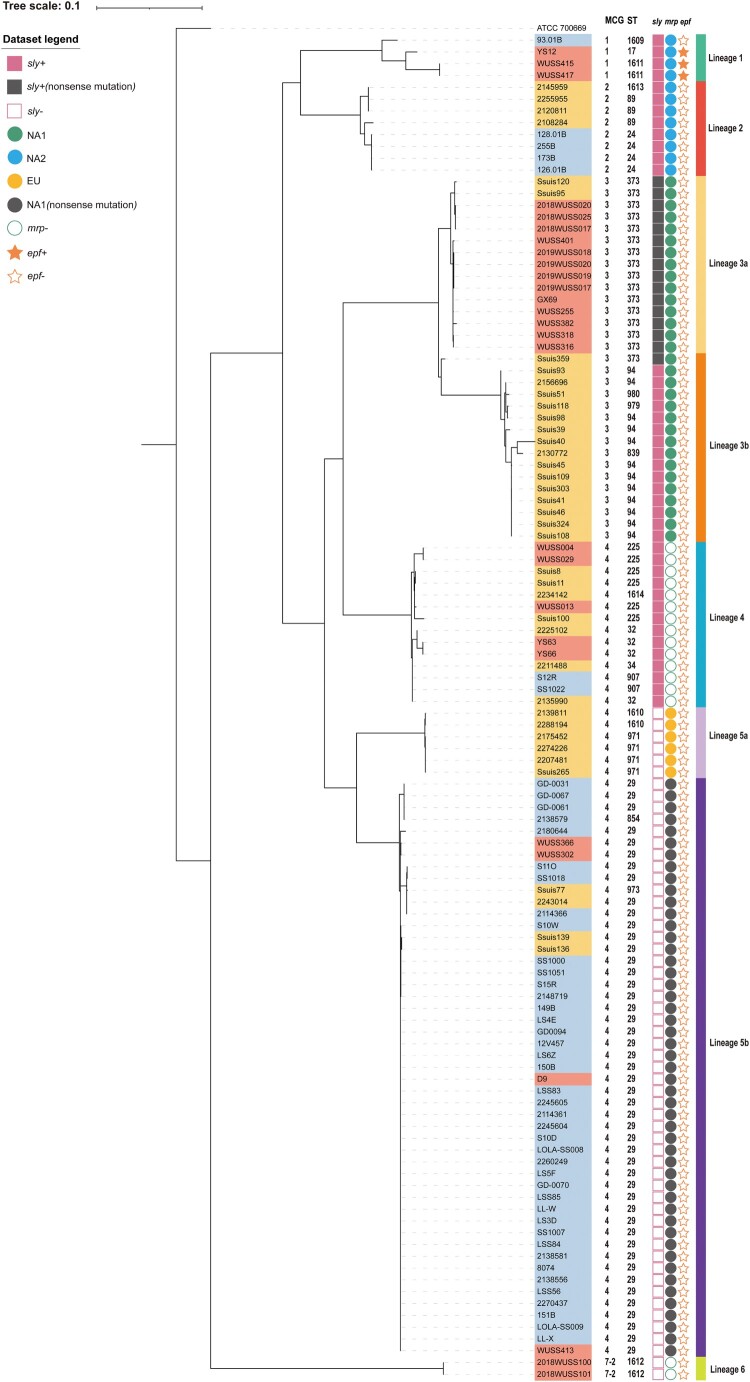
A maximum-likelihood phylogenetic tree of 114 *S. suis* serotype 7 genomes. The phylogenetic tree was constructed based on mutational SNPs differences across the whole core genome. The *S. pneumoniae* ATCC 700669 was used as an outgroup to root the tree. The strains were coloured based on the isolation regions, grey for Europe, orange-yellow for North America, and orange-red for China. The scale is given as the number of substitutions per variable site.

The 114 genomes were clustered into five MCG groups, including MCG groups 1, 2, 3, 4, and 7–2. MCG group 4 was predominant and included ST29 strains. It is noteworthy that genomes of MCG group 4 were composed of 10 STs and 69 genomes widely distributed in all nine countries. Five STs and 31 genomes were classified into MCG group 3, including the strain GX69 from the patient. Eight and four genomes were classified into MCG groups 2 and 1, respectively. Both of them contained 3 STs. Two ST1612 genomes were classified into MCG group 7–2 (Figure 1).

Based on the distribution of mutational SNPs in core genomes, 114 genomes were clustered into six lineages. Each MCG group consisted of one lineage, except for MCG group 4. Both Lineages 4 and 5 were composed of MCG group 4, whereas contained 14 and 55 genomes, respectively. Lineage 3 was divided into Lineages 3a and 3b. Interestingly, genomes of Lineage 3a and Lineage 3b were mainly from China and USA, respectively. Compared with Lineage 3a, composed of ST373 genomes, Lineage 3b was mainly composed of ST94 genomes. Lineages 5a and 5b were mainly composed of ST971 and ST29 genomes, respectively (Figure 1).

The difference in virulence among S. suis serotype 7 strains.

In order to evaluate the virulence level of *S. suis* serotype 7 population, we compared the survival level of C57BL/6 mice infected with *S. suis* highly pathogenic serotype 2 strain P1/7, strain GX69, and additional 21 serotype 7 representative strains. Most mice infected with *S. suis* serotype 7 strains showed obvious septic signs during the infection, such as rough hair coat, swollen eyes, weakness, and shivering. The apparent diversity in the survival curves of mice infected with *S. suis* serotype 7 strains were observed. A significant difference was observed in survival curves between mice infected with strains P1/7 and GX69 (*p* = .0002), which attributed to the differences in mortality at the early phase of the infection. Mice infected with P1/7 had a 20% survival rate at 12 h post-infection, while mice infected with strain GX69 had a 65% survival rate at the same time point. Notably, the survival levels of mice infected with strain GX69 dramatically decreased after 12 h post-infection. Its survival rate decreased to 10% at 24 h post-infection and was identical to that of strain P1/7 (Table 2, Figure S1A). Thus, strain GX69 possessed the capacity to initiate lethal infection in C57BL/6 mice and was classified as a virulent strain.

1. Among additional 21 serotype 7 representative strains, the mortalities of mice infected with eight *S. suis* serotype 7 strains at 96 h post-infection were less than 50%. These strains were classified as lowly virulent strains. Interestingly, half of them were isolated from diseased pigs.
  1. None of the mice infected with strains 128.01B, 173B, WUSS316, WUSS302, and 8074 died within the infection period (Table 2), even though strains 128.01B, 173B, and 8074 were isolated from diseased pigs.
  2. The survival mice infected with strains 126.01B (*p* < .0001), WUSS382 (*p* < .0001), and 2018WUSS100 (*p* < .0001) were significantly higher than that of mice infected with strain GX69 (Table 2). The strain 126.01B was isolated from diseased pig.
2. The survival levels of mice infected with remaining 13 *S. suis* serotype 7 representative strains were significantly higher than that of mice infected with strains P1/7. However, the mortalities of mice infected with these strains at 96 h post-infection reached or exceeded 80% (Table 2). These strains were classified as virulent strains. It is noteworthy that seven of them were isolated from healthy pigs.
  1. The survival curves of mice infected with YS63 (*p* < .0001), WUSS013 (*p* < .0001), and WUSS029 (*p* = .0021) were significantly different from that of mice infected strain GX69, since mice infected with three strain mainly died after 24 h post-infection (Table 2 and Figure S1B). Two strains YS63 and WUSS029 were isolated from healthy pigs.
  2. The survival curves of mice infected with 10 strains YS12 (*p* = .2817), WUSS415 (*p* = .7557), 93.01B (*p* = .0592), 2018WUSS020 (*p* = .2705), 2019WUSS020 (*p* = .8214), WUSS004 (*p* = .0838), WUSS413 (*p* = .2015), 149B (*p* = .7383), 150B (*p* = .3338), and 151B (*p* = .9834) were similar to that of mice infected with strain GX69 (Table 2 and Figure S1C). Therefore, they were classified as virulent strains. Among them, strains YS12, WUSS415, 2018WUSS020, 2019WUSS020, and WUSS413 were isolated from healthy pigs.

**Table 2.** The value of mortality and statistical comparison in the survival assay.

Lineage	Strains	The mean mortality of each infected group at different post-infection time points**^a^**									Virulence level**^b^**	*p* value**^c^**		
		6h	12h	18 h	24h	36h	48 h	60h	72h	96 h		Compare to P1/7 infected group	Compare to GX69 infected group	Compare to control group
lineage1	YS12	0 ± 0	50 ± 7	75 ± 4	100 ± 0	100 ± 0	100 ± 0	100 ± 0	100 ± 0	100 ± 0	V	.0009	.2817	<.0001
	WUSS415	0 ± 0	10 ± 7	85 ± 4	90 ± 7	90 ± 7	90 ± 7	90 ± 7	90 ± 7	90 ± 7	V	<.0001	.7557	<.0001
	93.01B	0 ± 0	0 ± 0	45 ± 32	90 ± 0	90 ± 0	95 ± 4	95 ± 4	95 ± 4	100 ± 0	V	<.0001	.0592	<.0001
lineage2	126.01B	0 ± 0	0 ± 0	0 ± 0	0 ± 0	0 ± 0	0 ± 0	0 ± 0	10 ± 0	20 ± 0	L	<.0001	<.0001	.1468
	128.01B	0 ± 0	0 ± 0	0 ± 0	0 ± 0	0 ± 0	0 ± 0	0 ± 0	0 ± 0	0 ± 0	L	<.0001	<.0001	>.9999
	173B	0 ± 0	0 ± 0	0 ± 0	0 ± 0	0 ± 0	0 ± 0	0 ± 0	0 ± 0	0 ± 0	L	<.0001	<.0001	>.9999
lineage3a	GX69	0 ± 0	35 ± 14	65 ± 7	90 ± 4	90 ± 7	90 ± 7	90 ± 7	90 ± 7	90 ± 7	V	.0002	/	<.0001
	WUSS316	0 ± 0	0 ± 0	0 ± 0	0 ± 0	0 ± 0	0 ± 0	0 ± 0	0 ± 0	0 ± 0	L	<.0001	<.0001	>.9999
	WUSS382	0 ± 0	0 ± 0	0 ± 0	0 ± 0	20 ± 0	30 ± 7	35 ± 11	40 ± 7	40 ± 7	L	<.0001	<.0001	.0262
	2018WUSS020	0 ± 0	23 ± 3	57 ± 7	70 ± 5	83 ± 3	83 ± 3	83 ± 3	83 ± 3	83 ± 3	V	<.0001	.2705	.0001
	2019WUSS020	0 ± 0	30 ± 7	70 ± 7	80 ± 0	80 ± 0	80 ± 0	80 ± 0	80 ± 0	80 ± 0	V	.0001	.8214	.0002
lineage4	YS63	0 ± 0	0 ± 0	0 ± 0	45 ± 11	65 ± 11	90 ± 7	90 ± 7	90 ± 7	90 ± 7	V	<.0001	<.0001	<.0001
	WUSS004	0 ± 0	23 ± 11	37 ± 12	70 ± 8	90 ± 5	93 ± 5	93 ± 5	93 ± 5	93 ± 5	V	<.0001	.0838	<.0001
	WUSS013	0 ± 0	0 ± 0	0 ± 0	0 ± 0	0 ± 0	10 ± 7	60 ± 21	85 ± 4	100 ± 0	V	<.0001	<.0001	<.0001
	WUSS029	0 ± 0	10 ± 7	15 ± 4	45 ± 18	75 ± 11	95 ± 4	95 ± 4	95 ± 4	95 ± 4	V	<.0001	.0021	<.0001
Lineage5b	WUSS302	0 ± 0	0 ± 0	0 ± 0	0 ± 0	0 ± 0	0 ± 0	0 ± 0	0 ± 0	0 ± 0	L	<.0001	<.0001	>.9999
	WUSS413	0 ± 0	20 ± 5	47 ± 7	83 ± 10	97 ± 3	100 ± 0	100 ± 0	100 ± 0	100 ± 0	V	<.0001	.2015	<.0001
	149B	0 ± 0	37 ± 14	50 ± 17	93 ± 3	100 ± 0	100 ± 0	100 ± 0	100 ± 0	100 ± 0	V	<.0001	.7383	<.0001
	150B	0 ± 0	0 ± 0	75 ± 3	90 ± 0	100 ± 0	100 ± 0	100 ± 0	100 ± 0	100 ± 0	V	<.0001	.3338	<.0001
	8074	0 ± 0	0 ± 0	0 ± 0	0 ± 0	0 ± 0	0 ± 0	0 ± 0	0 ± 0	0 ± 0	L	<.0001	<.0001	>.9999
	151B	0 ± 0	33 ± 12	60 ± 14	100 ± 0	100 ± 0	100 ± 0	100 ± 0	100 ± 0	100 ± 0	V	<.0001	.9834	<.0001
lineage6	2018WUSS100	0 ± 0	0 ± 0	0 ± 0	15 ± 9	30 ± 6	40 ± 6	40 ± 6	40 ± 6	45 ± 3	L	<.0001	<.0001	.0168
	P1/7	60 ± 7	80 ± 0	90 ± 0	90 ± 0	90 ± 0	90 ± 0	90 ± 0	90 ± 0	90 ± 0	H	/	.0002	<.0001
	Control	0 ± 0	0 ± 0	0 ± 0	0 ± 0	0 ± 0	0 ± 0	0 ± 0	0 ± 0	0 ± 0	/	<.0001	<.0001	/

^a^ The mortality represented as mean ± SED (calculated via the Kaplan–Meier method) at different post-infection time points was present.

^b^ H indicates highly virulent, *V* indicates virulent, and *L* indicates lowly virulent.

^c^ The survival curves of different infected groups were compared using Gehan–Breslow–Wilcoxon test.

Interestingly, all three tested strains from Lineage 1 were classified as virulent strains, while all strains from Lineages 2 and 6 belonged to lowly virulent strains. On the contrary, Lineages 3a, 4, and 5b contained both virulent and lowly virulent strains.

### Distribution of putative S. suis virulence-related genes

Only three genomes (YS12, WUSS415, and WUSS417) of Lineage 1 were positive for *epf*. Sixteen genomes from Lineages 4 and 6 were *mrp* gene negative. Most of the genomes (98/114, 86.0%) contained putative full-length *mrp* gene copies. Based on the variation in the central portion of the gene, *mrp* was grouped into three subtypes EU, NA1, and NA2 [38]. Subtype NA2 (*n* = 12) was only present in genomes of Lineages 1 and 2. Subtype EU (*n* = 6) was only found in genomes of Lineage 5a. All genomes of Lineage 3 and 5b harboured subtype NA1(*n* = 31). Compared with those of Lineage 3, *mrp* gene of Lineage 5b (*n* = 49) did not contain an intact open reading frame because of a frameshift mutation in 2.1 kb, which may result in the truncated MRP expression. *sly* gene was only present in genomes of Lineages 1, 2, 3, and 4. A premature stop codon was present in *sly* gene of ST373 genomes of Lineage 3 (Figure 1).

Genes *nadR*, *NisR*, *NisK*, *SalR*, and *SalK* were absent from all serotype 7 genomes. Genes *revS* and *ofs* were only present in genomes of Lineage 1. *sao* gene was widely distributed in serotype 7 genomes, except for genomes of Lineage 6. RD6 was present in strains WUSS415, WUSS417, and YS12, while the remaining RDs tested in the study were absent from all serotype 7 genomes.

### The distribution of AR genes in S. suis serotype 7 genomes

Thirteen genomes did not harbour any AR genes. Totally, 216 AR genes were present in the remaining 101 genomes. The AR genes belonged to six categories tetracycline, macrolides/lincosamides/streptogramin (MLS), lincosamide, aminoglycosides, trimethoprim, and chloramphenicol (Table 1).

#### The tetracyclines resistance genes

Ninety-three genomes carried tetracycline-resistant genes. Three types of tetracycline-resistant genes were found among them, consisting of *tet*(O), *tet*(M), and *tet*(W). *tet*(O) was the prevalent tetracycline-resistant gene and was present in 72 genomes. Eighteen genomes carried *tet*(M) gene, mainly from Lineage 5b (14/18). *tet*(W) gene was present in three genomes.

#### The MLS and lincosamide resistance genes

Three types of genes were found, consisting of genes *erm*(B), *lsa*C, and *lnu*B. The MLS resistance gene *erm*(B), encoding rRNA adenine N-6-methyltransferase, was prevalent and present in 72 genomes. The lincosamides-streptogramin A resistance gene *lsa*C was found in three genomes of Lineage 6, which simultaneously harboured lincosamide resistance gene *lnu*B.

#### The aminoglycosides resistance genes

Fifteen genomes carried aminoglycosides resistance genes, including streptomycin resistance gene *ant6ia* encoding aminoglycoside O-nucleotidylyltransferase (*n* = 13), kanamycin resistance gene *aph(3*′*)-IIIa* encoding aminoglycoside O-phosphotransferase (*n* = 9), spectinomycin resistance gene *ant9ia* encoding aminoglycoside 3′-phosphotransferase (*n* = 4), and gentamicin and kanamycin resistance gene *aac(6*′*)-Ie-aph(2*″*)-Ia* encoding aminoglycoside acetyltransferase (*n* = 2).

#### The trimethoprim and chloramphenicol resistance genes

Eleven *genomes* harboured trimethoprim resistance gene *dfrF* encoding dihydrofolate reductase. It is noteworthy that all genomes carried *dfrF* gene were from Lineage 6. One genome of Lineage 6 harboured chloramphenicol resistance gene *cat*-TC encoding chloramphenicol acetyltransferase.

### Antimicrobial susceptibility profiles of available strains

To investigate whether AR genes conferred resistance to host strains, we tested the antimicrobial susceptibility of available strains, including 25 Chinese and five Spanish strains, carrying genes responsible for resistance to tetracycline, erythromycin, clindamycin, streptomycin, kanamycin, or gentamycin. Thirty strains harboured tetracycline resistance genes were all resistant to tetracycline, with a MIC value between 12 and 128 μg/mL. Concomitant resistance to erythromycin and clindamycin was found in all strains (*n* = 25) carrying *erm*(B) gene due to the overlapping ribosomal binding sites of the two antibiotics. MIC values for both antibiotics were between 128 and >256 μg/mL. A high level of kanamycin (MICs > 256 μg/mL) resistance was found in four strains carrying *aph3-IIIa* gene. The MIC values of both gentamycin and kanamycin were >256 μg/mL in two strains carrying *aac(6*′*)-Ie-aph(2*″*)-Ia* gene. The MIC values of streptomycin were >1024 μg/mL in nine strains carrying the *ant6ia* gene (Table S1). The data confirmed that these AR genes conferred corresponding antibiotic resistance phenotypes to their host.

### AR genes associated with MGEs

**Figure 2.**
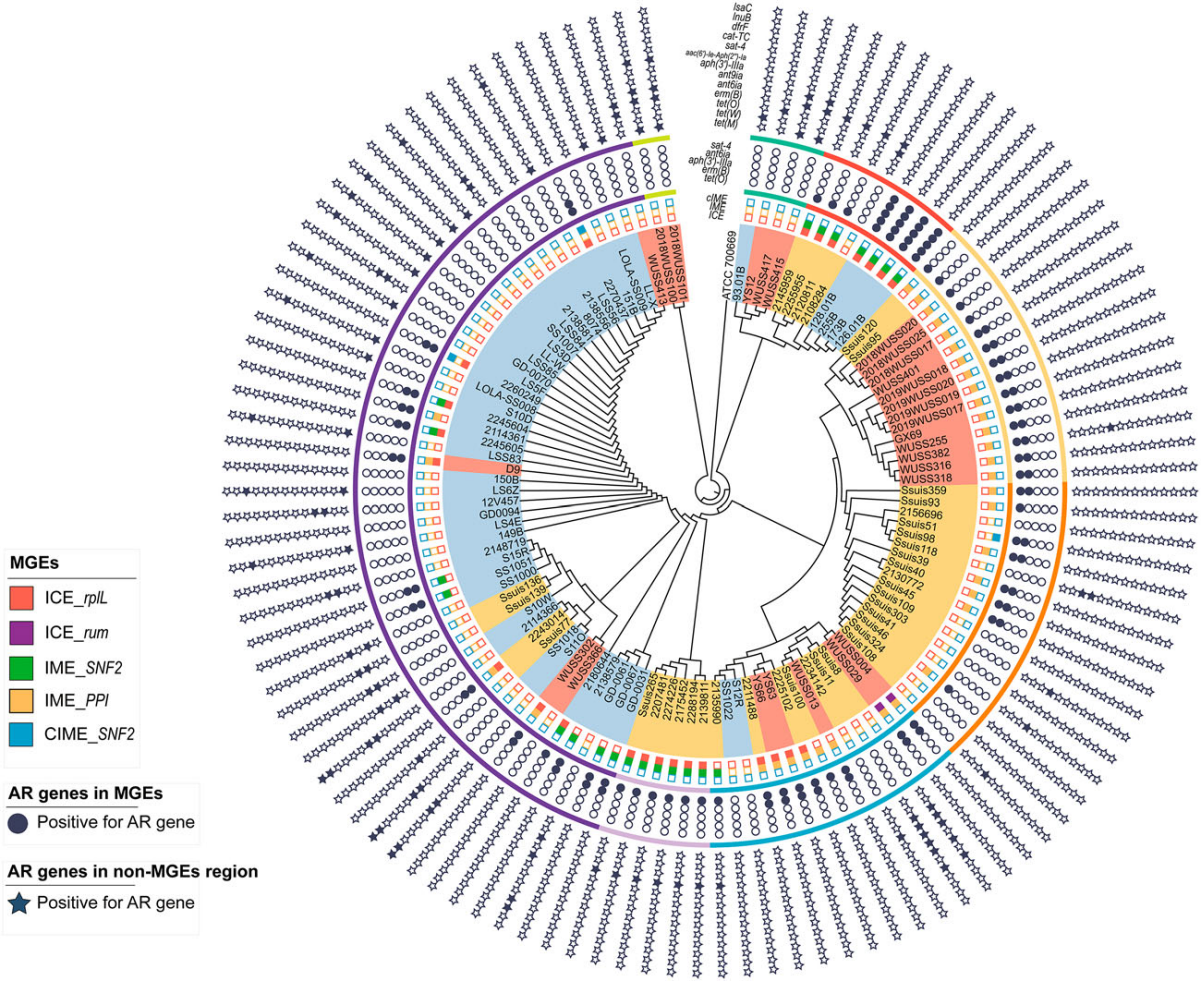
The distribution of AR genes in MGEs and non-MGEs regions of *S. suis* serotype 7 genomes. The inner circle is the distribution of ICEs, IMEs, and CIMEs in S. *suis* serotype 7 genomes. Each panel of boxes filled with different colors represents ICEs, IMEs, and CIMEs integrated into the different locus of corresponding genomes, and hollow boxes represent the absence of ICEs, IMEs, or CIMEs in corresponding genomes. The middle circle showed the AR genes carried by ICEs, IMEs, or CIMEs. Each filled circle represents the corresponding AR gene present on corresponding ICEs, IMEs, or CIMEs. The outer circle showed the AR genes located into non-MGEs regions in S. *suis* serotype 7 genomes. Each filled star represents the corresponding AR gene present on non-MGEs regions in corresponding genomes.

To investigate the mechanism to disseminate AR genes, the MGEs harbouring AR genes in *S. suis* serotype 7 genomes were predicted. Among 114 genomes, 27 ICEs, 56 IMEs, and three CIMEs (absent of the integrase and relaxase genes) with a complete sequence were detected. These ICEs were distributed in Lineages 2, 4, and 5, whereas IMEs were distributed in Lineages 2, 3, 4, and 5. Totally, 111 of 216 AR genes were present in these MGEs (Figure 2).

Twenty-five ICEs were inserted into *rplL* locus. All of them harboured a 15-bp *att* sequence 5′-TTATTTAAGAGTAAC-3′ in the flanking region. ICESsuWUSS029 and ICESsuWUSS004 were integrated into downstream of *rum* gene. Both of them harboured the 14-bp *att* sequences 5’-CACGTGGAGTGCGT-3′ and 5′-CATGTTGAAGTTGT-3′ in the 5′ and 3′ flanking regions, respectively. All ICEs were classified as Tn5252 group and harboured intact signature proteins VirB4, integrase, and canonical relaxase of the MobP family. Fifty-six AR genes resistant to tetracycline, MLS, and aminoglycosides were present in these ICEs (Figure 2).

The genes *SNF2* and *PPI* encoding a putative adenine-specific DNA methylase and a putative peptidylprolyl isomerase, respectively, are two specific insertion hot spots for integrating IMEs or CIMEs [32]. In the present study, 34 and 22 IMEs were integrated into the PPI and SNF2 genes, respectively. All three CIMEs were integrated into *SNF2* gene (Figure 2). All IMEs or CIMEs harboured an 11-bp inverted repeat 5′-TTTTGCGGACA-3′ in their flanking region. Interestingly, 25 IMEs and two CIMEs were integrated into ICEs and all AR genes in the ICEs were carried by these integrated IMEs and CIMEs. The remaining 31 IMEs and one CIMEs were located in non-ICE regions. Thirty-two *tet*(O) and 23 *erm*B genes were present in these IMEs and CIMEs. The integrases of all IMEs were identical and belonged to serine integrase type 3, regardless of their integration site. Meanwhile, the relaxases of the IMEs belonged to the MobV superfamily. Based on their integrase and relaxase types, all IMEs belonged to IME_Class_6. It is noteworthy that all AR genes responsible for resistance to aminoglycosides, trimethoprim, and chloramphenicol were not present in the above MGEs.

### Differences of cps gene clusters among strains

**Figure 3.**
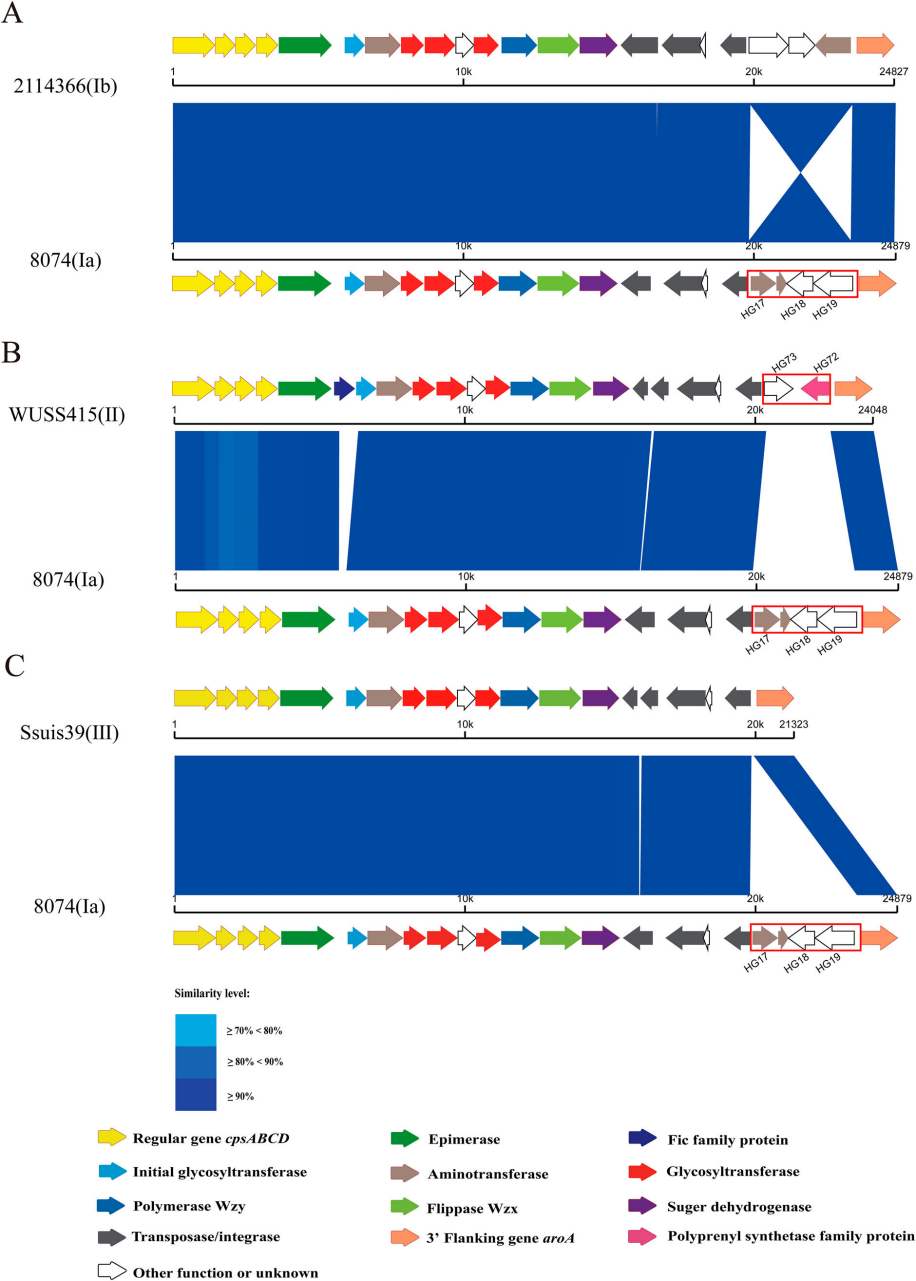
The schematic comparison of the *cps* gene cluster subtype Ia to that of Ib (A), II (B), and III (C). Each colored arrow represents the gene whose predicted function is shown in the blow panel. HG17, HG18, HG19, HG72, and HG73 genes are indicated. The *aroA* gene is located on the 3′ side of each locus. Regions of over 70% identity were marked by blue shading.

The *cps* gene clusters of 114 genomes were located between the *orfZ*-*orfX* region and the *aroA* gene, which belonged to pattern I-a [33]. Based on the variable presence of HG17 (Aspartate aminotransferase), HG18 (Tetratricopeptide repeat protein) and HG19 (Hypothetical protein) of the serotype 7 reference strain 8074 (GenBank accession No. BR001004.1), four subtypes of *cps* gene clusters were found among 114 genomes (Figure 3).

HG17, HG18, and HG19 were present in *cps* gene clusters of 84 genomes that belonged to *cps*7-I. The arrangement of HG17, HG18, and HG19 in *cps* gene clusters of 54 genomes similar to that of the serotype 7 reference strain 8074 was nominated as *cps*7-Ia, while the arrangement of them inverted in *cps* gene clusters of 30 genomes was nominated as *cps*7-Ib. *cps*7-Ib was mainly present in genomes from Lineage 5b (*n* = 11), Lineage 4 (*n* = 9) and Lineage 3a (*n* = 7).

Replacement of HG17, HG18, and HG19 by HG72 and HG73 was found in *cps* gene clusters of 23 genomes which were nominated as *cps*7-II. These genomes were distributed in Lineage 5b (*n* = 16), Lineage 2 (*n* = 4), and Lineage 1 (*n* = 3), from European countries and China.

HG17, HG18, and HG19 were absent in seven *cps* gene clusters which were nominated as *cps* 7-III. Five of them were from the USA and distributed in Lineage 5b, while two were from China and consisted of Lineage 6.

## Discussion

In the present study, we first reported a *S. suis* serotype 7 strain (GX69) isolated from a patient with septicemia complicated with pneumonia. The strain GX69 was ST373 and belonged to MCG group 3, whereas ST1 and ST7 are predominant in *S. suis* strains from patients [39], mainly clustered into MCG group 1 [26]. The genotype of *S. suis* classical virulence markers in strain GX69 was *mrp*^NA1^ *sly*^+^ *epf ^–^*, whereas the prevalent genotype of corresponding virulence markers in human strains was *mrp*^EU^ *sly*^+^ *epf^+^* or *mrp*^NA2^ *sly*^+^ *epf^+^*[9,40]. It is noteworthy that a premature stop codon was present in *sly* gene of strain GX69 and may result in the truncated SLY expression. To evaluate the virulence of strain GX69, the survival level was compared with that of highly pathogenic *S. suis* serotype 2 strain P1/7. Significant differences at the early phase of infection and the similarity at the middle phase of infection were observed between the two strains in the mouse infection model. Our result confirmed that GX69 was a virulent strain and possessed the capacity to initiate lethal infection, even though virulence-associated genes and RDs preferentially present in highly pathogenic *S. suis* serotype 2 strains were almost absent in strain GX69. We proposed that *S. suis* serotype 7 may be considered as a potential zoonotic pathotype, and further investigation of *S. suis* serotype 7 population is needed to improve the prevention and control strategies.

In the present study, *S. suis* serotype 7 population composed of 35 strains and 79 genomes of strains from 1999 to 2019 in nine countries was investigated. Twenty-two STs and five MCG groups were identified among *S. suis* serotype 7 genomes clustered into six lineages based on the distribution of mutational SNPs in the core genomes. Interestingly, since most predominant ST29, ST373, and ST94 were prevalent in respective regions, it suggests that the evolution of *S. suis* serotype 7 population was relevant to the geographical distribution. The evolutionary affinity between ST373 and ST94 was revealed in that they belonged to MCG group 3 and were clustered into Lineage 3. It is noteworthy that significant heterogeneity was observed within ST373 strains, which were clustered into three clades. Similar heterogeneity was previously reported in phylogenetic analysis of ST1[40], ST7 [9], and ST25 [41].

Based on the results of the survival assay using the C57BL/6 mouse model, the strain GX69 and over 60% additional representative strains tested (13/21) were classified as virulent strains. Among 13 virulent strains, the survival curves of ten *S. suis* serotype 7 representative strains were similar to that of strain GX69. In a recent study, 82.6% *S. suis* serotype 7 strains from North America were pathogenic based on the clinical information and site of isolation [20]. In the present study, seven strains isolated from healthy pigs were classified as virulent strains, and two of them belong to ST373, the same ST of strain GX69. Therefore, we proposed that the public health threat of *S. suis* serotype 7, especially those virulent ST373 strains, should not be ignored. Coincidentally, healthy pigs were reported to be a reservoir of strains with high virulence potential in humans [39,42]. Moreover, four strains isolated from diseased pigs were classified as lowly virulent. A correlation between the virulence level of strains and their origin (diseased or healthy pigs) could not be observed in the present study. A similar result was also reported in our previous study [43]. It should be noted that the presence of clinical signs in pigs may also depend on co-infection with some viral and bacterial pathogens [42]. Thus, the public health significance of strains may not be accurately evaluated only based on the clinical information of their host.

In the present study, three classical virulence markers *mrp*, *sly*, and *epf* were not critical virulence indicators of the *S. suis* serotype 7 strains. However, a significant correlation of genotypes and variations of three genes and their distribution in lineages was observed, suggesting that these genes correlate with the evolution of *S. suis* serotype 7 population rather than virulence. Most virulence-associated genes preferentially present in highly pathogenic *S. suis* serotype 2 strains were absent from all serotype 7 genomes. Previous studies also reported that these virulence markers studied in *S. suis* serotype 2 strains were not suitable as virulence markers for *S. suis* non-serotype 2 strains [3,12,44]. Thus, *S. suis* serotype 7 virulent strains may utilize a different pathogenesis strategy. Because of the high diversity of virulence levels within *S. suis* serotype 7 population, further studies are necessary to identify reliable virulence indicators of *S. suis* serotype 7 strains. Using multiple animal models to accurately pathotype ST373 strains combined with comparative genomic analysis of ST373 strains with different virulence levels may be feasible.

Six categories of AR genes are present in *S. suis* serotype 7 genomes. The predominant categories were tetracycline and MLS resistance genes. High rates of resistance to tetracycline, macrolide, lincosamide, and erythromycin have been reported in both human and pig isolates of *S. suis* in the last 20 years [45–49]. Tetracycline, lincosamide, and macrolide are used extensively for therapy and metaphylaxis in the swine industry [32,50,51], contributing to the emergence and spread of associated resistance. The most prevalent tetracycline resistance gene was *tet*(O). This is different from what was previously reported for serotype 2 strains, which have been shown to mainly carry *tet*(M) and *tet*(W) [45,48].

Previous studies have shown that MGEs play a significant role in the horizontal transfer of AR genes in *S. suis* [31,32]. Twenty-seven ICEs carrying AR genes were found in *S. suis* serotype 7 genomes, although intact prophages carrying AR genes were not detected. Conversely, AR genes in *S. suis* serotype 31 population were majorly present in prophages [12]. In the present study, two types of DNA cargo of IMEs and CIMEs with AR genes were integrated into genes *SNF2* or *PPI* of all ICEs. Similar IMEs were also integrated into *SNF2* gene of ICESsuZJ20091101-1(KX077882.1), ICESsuLP081102 (KX077885.1), ICESsuJH1301 (KX077887.1) [31], and ICESsD9 [52]. A similar CIME was also inserted into the same integration site of ICESsuBSB6 [53]. Likely, the exchange, acquisition, and deletion of the IME/CIME module may contribute to the evolution of ICEs. In the present study, all AR genes in ICEs were carried by these IMEs and CIMEs. Moreover, IMEs and CIMEs carrying AR genes were also present in non-ICE regions of additional 32 *S. suis* serotype 7 genomes.

IMEs were reported to be more widespread than ICEs in *S. suis* [32]. In this work, IMEs were also found to be highly prevalent in *S. suis* serotype 7 genomes. Over 50% AR genes identified in serotype 7 genomes were present in IMEs. IMEs mainly carried tetracycline, erythromycin, and lincosamide resistance genes. We propose that IMEs may play a critical role in the horizontal transfer of these AR genes in *S. suis* serotype 7. Interestingly, the proportion of genome carrying IMEs was higher in Lineages 2, 3a, 4, and 5a. Our data indicated that the transmission patterns of AR genes might be related to the evolution of serotype 7 population. CIMEs are decayed IMEs, which are *cis*-mobilizable elements without integration and relaxase genes but with *attL* and *attR* sites. CIMEs carrying *tet*(O) and *ermB* genes were found in two ICEs and non-ICE region of one additional genome. Further study is needed to investigate the role of CIMEs in the transmission of AR genes.

Finally, different organizations of *cps* loci were observed among *S. suis* serotype 7 population. These differences can be attributed to the variable presence of HG17, HG18, and HG19. The function of HG17 was related to aminotransferase, while the functions of both HG18 and HG19 were ATP-binding proteins. HG17, HG18, and HG19 existed widely in *cps* gene clusters of *S. suis* serotypes 4, 5, 17, 18, 19, and 23 reference strains. It is noteworthy that HG17, HG18, and HG19 were also inverted in *cps* gene clusters of *S. suis* serotypes 17 and 23 reference strains. Among 23 *cps* gene clusters of *S. suis* serotype 7 genomes, HG17, HG18 and HG19 were replaced by HG72 and HG73. The functions of HG72 and HG73 were related to carboxyvinyltransferase and unknown, respectively. HG72 and HG73 also existed in *cps* gene clusters of *S. suis* serotypes 11 and 30 reference strains. Based on the agglutination test results, the capsular antigenic phenotype was not affected by the variable presence of HG17, HG18, and HG19. Therefore, these HGs may not be involved in the forming of serotype 7-specific epitopes. The subtype *cps7*-I was most predominant among *S. suis* serotype 7 population and dispersed in different lineages and geographical regions. On the other hand, subtype *cps*7-II was majorly present in strains from Europe (such as UK and Spain), while it was absent in strains from North America. Different *cps* subtypes may enhance the fitness of corresponding host strains in specific environments.

In conclusion, our data confirmed *S. suis* serotype 7 is a non-negligible pathotype and deepened the understanding of *S. suis* serotype 7 population. Geographically dependent characteristics were revealed in the evolution of *S. suis* serotype 7 population. Our study provided valuable information for the improved surveillance of *S. suis* serotype 7 strains. Further studies are needed to identify the virulence indicators to predict the public health significance of *S. suis* serotype 7 strains.

## Supplementary Material

Figure_S1.tif

Clean_copy_of_suppelementary_materials.docx

## References

[1] Goyette-Desjardins G, Auger JP, Xu J, et al. *Streptococcus suis*, an important pig pathogen and emerging zoonotic agent-an update on the worldwide distribution based on serotyping and sequence typing. Emerg Microb Infect. 2014 Jun;3(6):e45. 10.1038/emi.2014.45 PMC4078792 26038745

[2] Bojarska A, Janas K, Pejsak Z, et al. Diversity of serotypes and new *cps* loci variants among *Streptococcus suis* isolates from pigs in Poland and Belarus. Vet Microbiol. 2020;240:108534. 31902504 10.1016/j.vetmic.2019.108534

[3] Huang J, Liu X, Chen H, et al. Identification of six novel capsular polysaccharide loci (NCL) from *Streptococcus suis* multidrug resistant non-typeable strains and the pathogenic characteristic of strains carrying new NCLs. Transbound Emerg Dis. 2019;66(2):995–1003. 30676694 10.1111/tbed.13123

[4] Zheng H, Qiu X, Roy D, et al. Genotyping and investigating capsular polysaccharide synthesis gene loci of non-serotypeable *Streptococcus suis* isolated from diseased pigs in Canada. Vet Res. 2017;48(1):10. 28219415 10.1186/s13567-017-0417-6 PMC5322794

[5] Qiu X, Bai X, Lan R, et al. Novel capsular polysaccharide loci and new diagnostic tools for high-throughput capsular gene typing in *Streptococcus suis*. Appl Environ Microbiol. 2016;82(24):7102–7112. 27694240 10.1128/AEM.02102-16 PMC5118921

[6] Huong VT, Ha N, Huy NT, et al. Epidemiology, clinical manifestations, and outcomes of *Streptococcus suis* infection in humans. Emerg Infect Dis. 2014;20(7):1105–1114. 24959701 10.3201/eid2007.131594 PMC4073838

[7] Yu H, Jing H, Chen Z, et al. Human *Streptococcus suis* outbreak, Sichuan, China. Emerg Infect Dis. 2006;12(6):914–920. 16707046 10.3201/eid1206.051194 PMC3373052

[8] Ye C, Zhu X, Jing H, et al. *Streptococcus suis* sequence type 7 outbreak, Sichuan, China. Emerg Infect Dis. 2006;12(8):1203–1208. 16965698 10.3201/eid1208.060232 PMC3291228

[9] Wang M, Du P, Wang J, et al. Genomic Epidemiology of *Streptococcus suis* sequence type 7 sporadic infections in the Guangxi Zhuang autonomous region of China. Pathogens. 2019;8(4). 10.3390/pathogens8040187 PMC6963630 31614790

[10] Schultsz C, Jansen E, Keijzers W, et al. Differences in the population structure of invasive *Streptococcus suis* strains isolated from pigs and from humans in The Netherlands. PLoS One. 2012;7(5):e33854. 22563452 10.1371/journal.pone.0033854 PMC3341392

[11] Kerdsin A, Hatrongjit R, Gottschalk M, et al. Emergence of *Streptococcus suis* serotype 9 infection in humans. J Microbiol Immunol Infect. 2017;50(4):545–546. 26362754 10.1016/j.jmii.2015.06.011

[12] Wang X, Sun J, Bian C, et al. The population structure, antimicrobial resistance, and pathogenicity of *Streptococcus suis cps*31. Vet Microbiol. 2021;259:109149. 34147764 10.1016/j.vetmic.2021.109149

[13] Prufer TL, Rohde J, Verspohl J, et al. Molecular typing of *Streptococcus suis* strains isolated from diseased and healthy pigs between 1996-2016. PLoS One. 2019;14(1):e0210801. 30653570 10.1371/journal.pone.0210801 PMC6336254

[14] Unterweger C, Baums CG, Hocher M, et al. [Clinical situation, diagnosis and prevention of a *Streptococcus suis* serotype 7 problem on a farm]. Berl Munch Tierarztl Wochenschr. 2014 May-Jun;127(5–6):194–201. 24881269

[15] Nutravong T, Angkititrakul S, Jiwakanon N, et al. Identification of major *Streptococcus suis* serotypes 2, 7, 8 and 9 isolated from pigs and humans in upper northeastern Thailand. Southeast Asian J Trop Med Public Health. 2014 Sep;45(5):1173–1181. 25417521

[16] Tian Y, Aarestrup FM, Lu CP. Characterization of *Streptococcus suis* serotype 7 isolates from diseased pigs in Denmark. Vet Microbiol. 2004;103(1–2):55–62. 15381266 10.1016/j.vetmic.2004.07.009

[17] Tarradas C, Perea A, Vela AI, et al. Distribution of serotypes of *Streptococcus suis* isolated from diseased pigs in Spain. Vet Rec. 2004;154(21):665–666. 15198317 10.1136/vr.154.21.665

[18] MacLennan M, Foster G, Dick K, et al. *Streptococcus suis* serotypes 7, 8 and 14 from diseased pigs in Scotland. Vet Rec. 1996;139(17):423–424. 8923718 10.1136/vr.139.17.423

[19] Rieckmann K, Seydel A, Szewczyk K, et al. *Streptococcus suis cps*7: an emerging virulent sequence type (ST29) shows a distinct, IgM-determined pattern of bacterial survival in blood of piglets during the early adaptive immune response after weaning. Vet Res. 2018;49(1):48. 29903042 10.1186/s13567-018-0544-8 PMC6003162

[20] Estrada AA, Gottschalk M, Rossow S, et al. Serotype and genotype (Multilocus sequence type) of *Streptococcus suis* isolates from the United States serve as predictors of pathotype. J Clin Microbiol. 2019;57(9). 10.1128/JCM.00377-19 PMC6711919 31243086

[21] Ishida S, Tien le HT, Osawa R, et al. Development of an appropriate PCR system for the reclassification of *Streptococcus suis*. J Microbiol Meth. 2014;107:66–70. 10.1016/j.mimet.2014.09.003 25229648

[22] Bai X, Liu Z, Ji S, et al. Simultaneous detection of 33 *Streptococcus suis* serotypes using the luminex xTAG(R) assay. J Microbiol Meth. 2015;117:95–99. 10.1016/j.mimet.2015.07.018 26210040

[23] Chatellier S, Harel J, Zhang Y, et al. Phylogenetic diversity of *Streptococcus suis* strains of various serotypes as revealed by 16S rRNA gene sequence comparison. Int J Syst Bacteriol. 1998;48(Pt 2):581–589. 9731300 10.1099/00207713-48-2-581

[24] Tien LHT, Nishibori T, Nishitani Y, et al. Reappraisal of the taxonomy of *Streptococcus suis* serotypes 20, 22, 26, and 33 based on DNA-DNA homology and sodA and recN phylogenies. Vet Microbiol. 2013;162(2-4):842–849. 23245487 10.1016/j.vetmic.2012.11.001

[25] Wang J, Yi X, Liang P, et al. Investigation of the genomic and pathogenic features of the potentially zoonotic *Streptococcus parasuis*. Pathogens. 2021 Jul 2;10(7):1–18. 10.3390/pathogens10070834 PMC8308872 34357984

[26] Chen C, Zhang W, Zheng H, et al. Minimum core genome sequence typing of bacterial pathogens: a unified approach for clinical and public health microbiology. J Clin Microbiol. 2013;51(8):2582–2591. 23720795 10.1128/JCM.00535-13 PMC3719615

[27] Ye C, Zheng H, Zhang J, et al. Clinical, experimental, and genomic differences between intermediately pathogenic, highly pathogenic, and epidemic *Streptococcus suis*. J Infect Dis. 2009;199(1):97–107. 19016627 10.1086/594370

[28] Zheng H, Du P, Qiu X, et al. Genomic comparisons of *Streptococcus suis* serotype 9 strains recovered from diseased pigs in Spain and Canada. Vet Res. 2018;49(1):1. 29316972 10.1186/s13567-017-0498-2 PMC5759227

[29] Zheng X, Zheng H, Lan R, et al. Identification of genes and genomic islands correlated with high pathogenicity in *Streptococcus suis* using whole genome tiling microarrays. PLoS One. 2011;6(3):e17987. 21479213 10.1371/journal.pone.0017987 PMC3068143

[30] Hu Y, Yang X, Qin J, et al. Metagenome-wide analysis of antibiotic resistance genes in a large cohort of human gut microbiota. Nature Commun. 2013;4:2151. 23877117 10.1038/ncomms3151

[31] Huang J, Ma J, Shang K, et al. Evolution and diversity of the antimicrobial resistance associated mobilome in *Streptococcus suis*: A probable mobile genetic elements reservoir for other streptococci. Front Cell Infect Microbiol. 2016;6(118):1–14. 27774436 10.3389/fcimb.2016.00118 PMC5053989

[32] Libante V, Nombre Y, Coluzzi C, et al. Chromosomal conjugative and mobilizable elements in *Streptococcus suis*: major actors in the spreading of antimicrobial resistance and bacteriocin synthesis genes. Pathogens. 2020;9(1):1–23. 10.3390/pathogens9010022 PMC7168690 31881744

[33] Okura M, Takamatsu D, Maruyama F, et al. Genetic analysis of capsular polysaccharide synthesis gene clusters from all serotypes of *Streptococcus suis*: potential mechanisms for generation of capsular variation. Appl Environ Microbiol. 2013;79(8):2796–2806. 23416996 10.1128/AEM.03742-12 PMC3623174

[34] Holden MT, Hauser H, Sanders M, et al. Rapid evolution of virulence and drug resistance in the emerging zoonotic pathogen *Streptococcus suis*. PLoS One. 2009;4(7):e6072. 19603075 10.1371/journal.pone.0006072 PMC2705793

[35] Lachance C, Gottschalk M, Gerber PP, et al. Exacerbated type II interferon response drives hypervirulence and toxic shock by an emergent epidemic strain of *Streptococcus suis*. Infect Immun. 2013;81(6):1928–1939. 23509145 10.1128/IAI.01317-12 PMC3676015

[36] Lachance C, Segura M, Gerber PP, et al. Toll-like receptor 2-independent host innate immune response against an epidemic strain of *Streptococcus suis* that causes a toxic shock-like syndrome in humans. PLoS One. 2013;8(5):e65031. 23724118 10.1371/journal.pone.0065031 PMC3665724

[37] Marie J, Morvan H, Berthelot-Herault F, et al. Antimicrobial susceptibility of *Streptococcus suis* isolated from swine in France and from humans in different countries between 1996 and 2000. J Antimicrob Chemother. 2002;50(2):201–209. 12161400 10.1093/jac/dkf099

[38] Fittipaldi N, Fuller TE, Teel JF, et al. Serotype distribution and production of muramidase-released protein, extracellular factor and suilysin by field strains of *Streptococcus suis* isolated in the United States. Vet Microbiol. 2009;139(3-4):310–317. 19596529 10.1016/j.vetmic.2009.06.024

[39] Dong X, Chao Y, Zhou Y, et al. The global emergence of a novel *Streptococcus suis* clade associated with human infections. EMBO Mol Med. 2021;13(7):e13810. 34137500 10.15252/emmm.202013810 PMC8261479

[40] Callejo R, Zheng H, Du P, et al. *Streptococcus suis* serotype 2 strains isolated in Argentina (South America) are different from those recovered in North America and present a higher risk for humans. JMM Case Rep. 2016;3(5):e005066. 28348788 10.1099/jmmcr.0.005066 PMC5343146

[41] Athey TB, Teatero S, Takamatsu D, et al. Population structure and antimicrobial resistance profiles of *Streptococcus suis* serotype 2 sequence type 25 strains. PLoS One. 2016;11(3):e0150908. 26954687 10.1371/journal.pone.0150908 PMC4783015

[42] Obradovic MR, Segura M, Segales J, et al. Review of the speculative role of co-infections in *Streptococcus suis*-associated diseases in pigs. Vet Res. 2021;52(1):49. 33743838 10.1186/s13567-021-00918-w PMC7980725

[43] Zheng H, Lan R, Zheng X, et al. Comparative genomic hybridization identifies virulence differences in *Streptococcus suis*. PLoS One. 2014;9(2):e87866. 24503649 10.1371/journal.pone.0087866 PMC3913679

[44] Dong W, Zhu Y, Ma Y, et al. Multilocus sequence typing and virulence genotyping of *Streptococcus suis* serotype 9 isolates revealed high genetic and virulence diversity. FEMS Microbiol Lett. 2017;364(22):1–8. 10.1093/femsle/fnx192 29029051

[45] Palmieri C, Varaldo PE, Facinelli B. *Streptococcus suis*, an emerging drug-resistant animal and human pathogen. Front Microbiol. 2011;2:235. 22275909 10.3389/fmicb.2011.00235 PMC3223616

[46] Princivalli MS, Palmieri C, Magi G, et al. Genetic diversity of *Streptococcus suis* clinical isolates from pigs and humans in Italy (2003-2007). Euro Surveill. 2009;14(33):1–7. 10.2807/ese.14.33.19310-en 19712640

[47] Zhang C, Ning Y, Zhang Z, et al. In vitro antimicrobial susceptibility of *Streptococcus suis* strains isolated from clinically healthy sows in China. Vet Microbiol. 2008;131(3-4):386–392. 18499362 10.1016/j.vetmic.2008.04.005

[48] Ye C, Bai X, Zhang J, et al. Spread of *Streptococcus suis* sequence type 7, China. Emerg Infect Dis. 2008;14(5):787–791. 18439362 10.3201/eid1405.070437 PMC2600270

[49] Wisselink HJ, Veldman KT, Van den Eede C, et al. Quantitative susceptibility of *Streptococcus suis* strains isolated from diseased pigs in seven European countries to antimicrobial agents licensed in veterinary medicine. Vet Microbiol. 2006;113(1–2):73–82. 16387456 10.1016/j.vetmic.2005.10.035

[50] Xu Z, Xie J, Peters BM, et al. Longitudinal surveillance on antibiogram of important gram-positive pathogens in southern China, 2001 to 2015. Microb Pathog. 2017;103:80–86. 27894963 10.1016/j.micpath.2016.11.013

[51] Seitz M, Valentin-Weigand P, Willenborg J. Use of antibiotics and antimicrobial resistance in veterinary medicine as exemplified by the swine pathogen *Streptococcus suis*. Curr Top Microbiol Immunol. 2016;398:103–121. 27738916 10.1007/82_2016_506

[52] Huang K, Song Y, Zhang Q, et al. Characterisation of a novel integrative and conjugative element ICESsD9 carrying erm(B) and tet(O) resistance determinants in *Streptococcus suis*, and the distribution of ICESsD9-like elements in clinical isolates. J Global Antimicrob Resis. 2016;7:13–18. 10.1016/j.jgar.2016.05.008 27531000

[53] Huang J, Chen L, Li D, et al. Emergence of a vanG-carrying and multidrug resistant ICE in zoonotic pathogen *Streptococccus suis*. Vet Microbiol. 2018;222:109–113. 30080664 10.1016/j.vetmic.2018.07.008

