# Supplementary figures and images for: Genomic and pathogenic investigations of Streptococcus suis serotype 7 population derived from a human patient and pigs
Source: Emerg Microbes Infect. 2021 Oct 17;10(1):1960–74. doi: 10.1080/22221751.2021.1988725 (PMC8525962; doi:10.1080/22221751.2021.1988725)

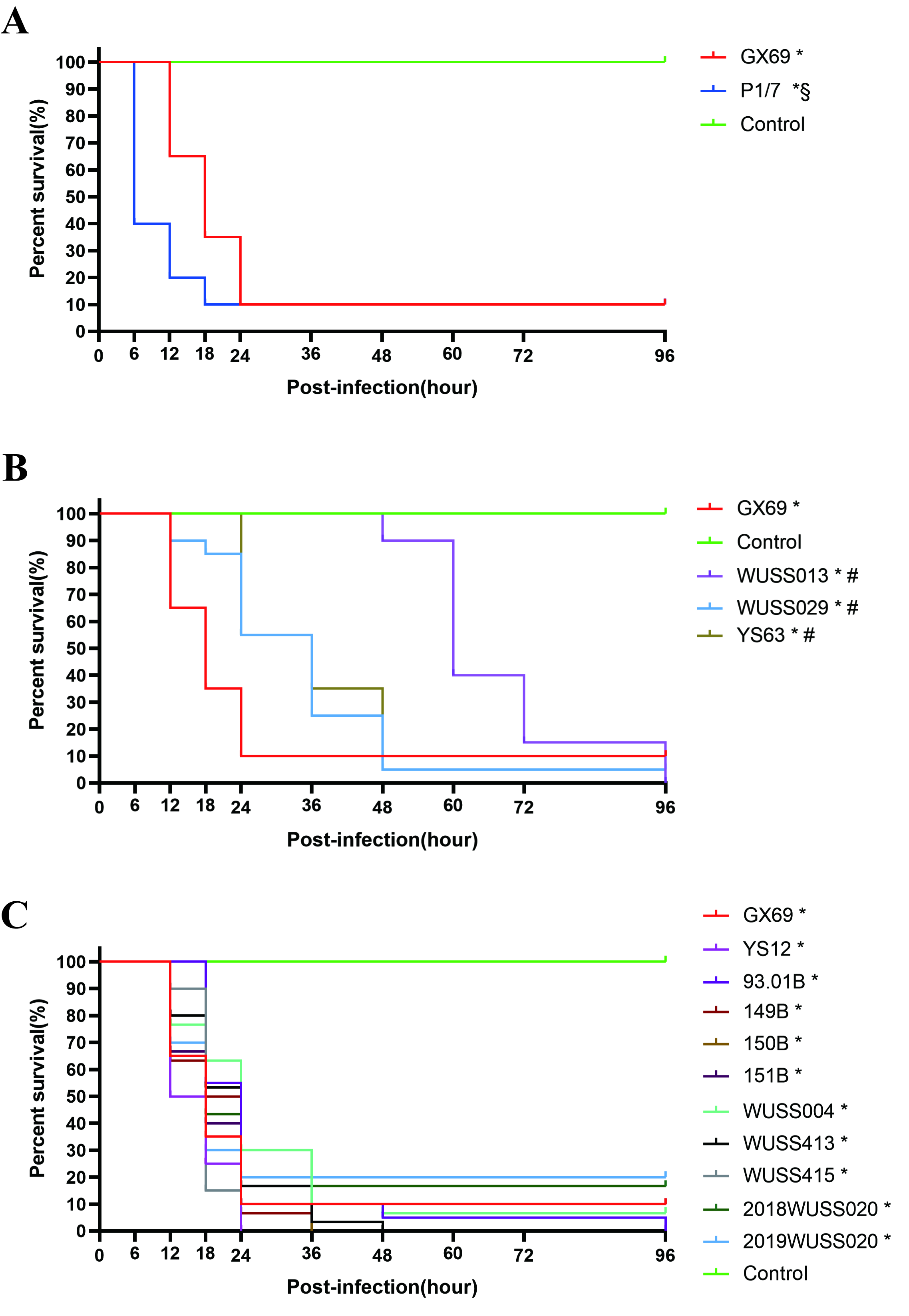

Supplement: Figure_S1.tif [file TEMI_A_1988725_SM2721.tif]
