# Supplementary material for: Genomic and pathogenic investigations of Streptococcus suis serotype 7 population derived from a human patient and pigs
Source: Emerg Microbes Infect. 2021 Oct 17;10(1):1960–74. doi: 10.1080/22221751.2021.1988725 (PMC8525962; doi:10.1080/22221751.2021.1988725)
Supplement: Clean_copy_of_suppelementary_materials.docx [file TEMI_A_1988725_SM2720.docx]

Supplemental Table 1. MIC values of antimicrobial agents for the available strains.

| Strains | MIC value of *S. suis* serotype 7 strains (μg/ml) | | | | | | |
| --- | --- | --- | --- | --- | --- | --- | --- |
|  | Clindamycin | Erythromycin | Azithromycin | Tetracycline | Gentamycin | Kanamycin | Streptomycin |
|  | Breakpoints＞1 | Breakpoints＞1 | Breakpoints＞2 | Breakpoints＞8 | Breakpoints＞250 | Breakpoints＞250 | Breakpoints＞250 |
| WUSS004 | ＞256 | 128 | 256 | 32 | / | / | / |
| WUSS013 | 128 | 128 | 256 | 64 | / | / | / |
| WUSS029 | 256 | 256 | 256 | 64 | / | / | / |
| WUSS255 | 128 | 128 | 128 | 32 | / | / | / |
| WUSS302 | 128 | 256 | 256 | 128 | / | / | / |
| WUSS316 | ＞256 | ＞256 | ＞256 | 32 | / | / | / |
| WUSS318 | 128 | ＞256 | 128 | 32 | / | / | / |
| WUSS382 | 128 | ＞256 | 256 | 32 | / | / | ＞1024 |
| WUSS401 | 128 | 128 | 128 | 32 | / | / | / |
| WUSS413 | 128 | ＞256 | ＞256 | 64 | / | / | / |
| WUSS415 | 128 | 128 | 256 | 12 | / | / | ＞1024 |
| WUSS417 | 128 | 256 | 256 | 12 | / | / | ＞1024 |
| 2018WUSS017 | / | / | / | 64 | / | / | / |
| 2018WUSS020 | / | / | / | 32 | / | / | / |
| 2018WUSS025 | / | / | / | 32 | / | / | / |
| 2018WUSS100 | 128 | ＞256 | ＞256 | 64 | ＞256 | ＞256 | ＞1024 |
| 2018WUSS101 | 256 | ＞256 | ＞256 | 128 | ＞256 | ＞256 | ＞1024 |
| 2019WUSS017 | ＞256 | ＞256 | ＞256 | 32 | / | / | / |
| 2019WUSS018 | ＞256 | ＞256 | ＞256 | 32 | / | / | / |
| 2019WUSS019 | ＞256 | ＞256 | ＞256 | 16 | / | / | / |
| 2019WUSS020 | ＞256 | ＞256 | ＞256 | 64 | / | / | / |
| YS12 | / | / | / | 12 | / | / | / |
| YS63 | ＞256 | ＞256 | ＞256 | 12 | / | / | / |
| YS66 | ＞256 | ＞256 | ＞256 | 12 | / | / | / |
| GX69 | ＞256 | ＞256 | ＞256 | 12 | / | / | / |
| 93.01B | / | / | / | 12 | / | / | / |
| 126.01B | ＞256 | ＞256 | ＞256 | 24 | / | ＞256 | ＞1024 |
| 128.01B | ＞256 | ＞256 | ＞256 | 16 | / | ＞256 | ＞1024 |
| 173B | ＞256 | ＞256 | ＞256 | 24 | / | ＞256 | ＞1024 |
| 255B | ＞256 | ＞256 | ＞256 | 24 | / | ＞256 | ＞1024 |


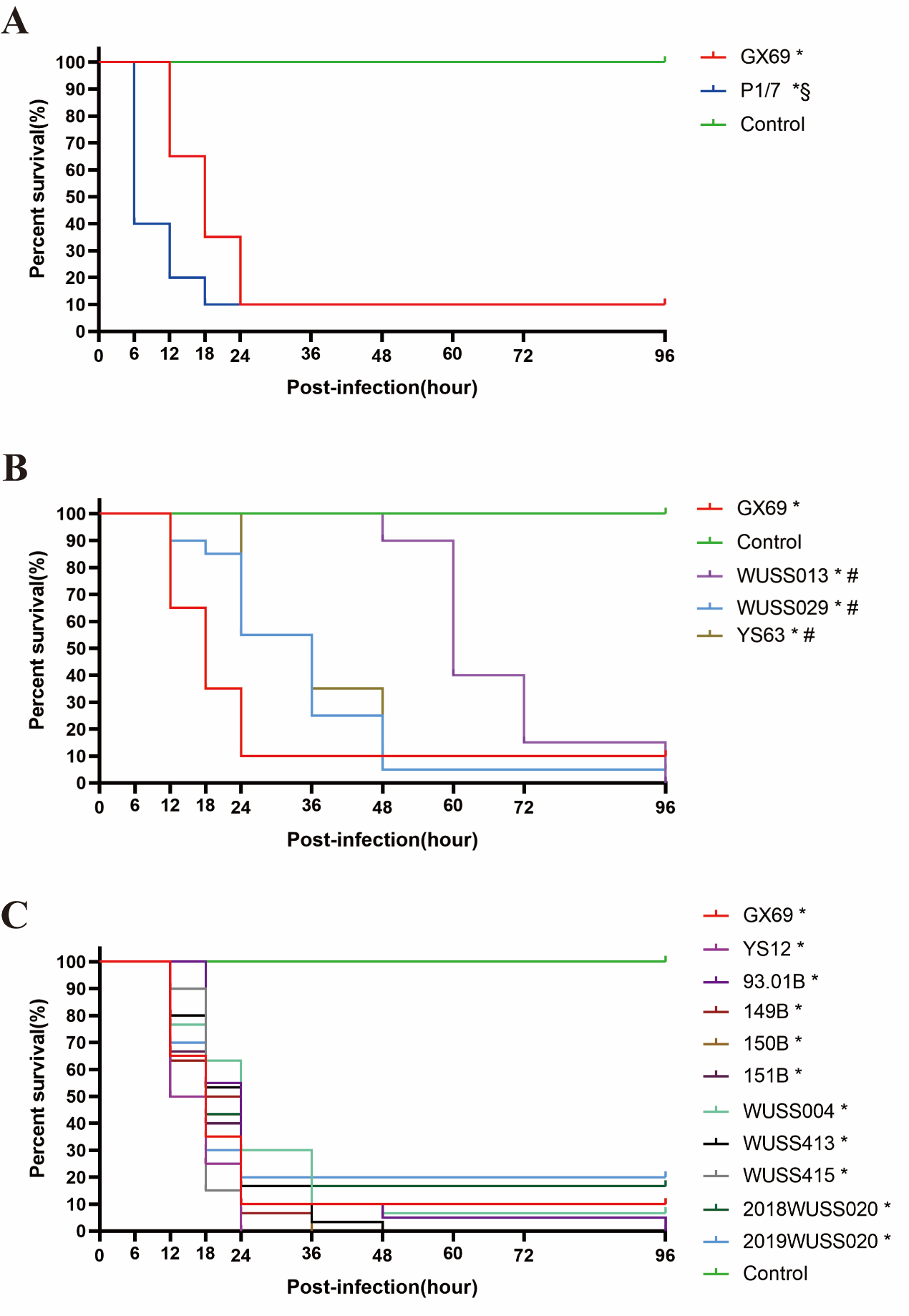


**Supplemental Figure 1. The survival curves of mice infected with *S. suis* serotype 7 virulent strains.** The mice in infection groups were infected with the dose of 5 × 10^7^ CFU per mouse. The mice in the control group were injected with PBS only. The experiments were performed independently at least twice for each strain. The mean survival rate was calculated by the Kaplan-Meier method at 12 h, 18 h, 24 h, 36 h, 48 h, 60h, 72 h, and 96 h post-infection. The survival curves were compared using Gehan-Breslow-Wilcoxon test. *p*＜0.05 was considered to be statistically different. A. The comparison of the survival curves of mice injected with strains P1/7, GX69, and PBS. B. The comparison of the survival curves of mice injected with strains YS63, WUSS013, WUSS029, GX69, and PBS. C. The comparison of the survival curves of mice injected with strains YS12, WUSS415, 93.01B, 2018WUSS020, 2019WUSS020, WUSS004, WUSS413, 149B, 150B 151B, GX69, and PBS. * Survival levels of mice from infection groups were statistically lower than that of mice from the control group. § Survival levels of mice infected with strains were statistically lower than that of mice infected with GX69. # Survival levels of mice infected with strains were statistically higher than that of mice infected with GX69.
